# Community perception and knowledge of cystic echinococcosis in the High Atlas Mountains, Morocco

**DOI:** 10.1186/s12889-018-6372-y

**Published:** 2019-01-28

**Authors:** Séverine Thys, Hamid Sahibi, Sarah Gabriël, Tarik Rahali, Pierre Lefèvre, Abdelkbir Rhalem, Tanguy Marcotty, Marleen Boelaert, Pierre Dorny

**Affiliations:** 1 Department of Public Health, Institute of Tropical Medicine, Nationalestraat 155, 2000 Antwerp, Belgium; 2 Department of Virology, Parasitology, and Immunology, Faculty of Veterinary Medicine, Ghent University, Salisburylaan 133, 9820 Merelbeke, Belgium; 3 Department of Pathology and Veterinary Public Health, Agro-Veterinary Institute Hassan II (IAV), Madinat Al Irfane. BP 6202-Instituts, 10101 Rabat, Morocco; 4 Department of Veterinary Public Health and Food Safety, Faculty of Veterinary Medicine, Ghent University, Salisburylaan 133, 9820 Merelbeke, Belgium; 5 Department of Biology, Faculty of Sciences of Rabat, University Mohamed V, 4 Avenue Ibn Battouta, B.P. 1014 Rabat, RP Morocco; 6 International Health, Brussels, Belgium; 7 Faculty of Science, University of Namur, Rue de Bruxelles 61, 5000 Namur, Belgium; 8 Department of Biomedical Sciences, Institute of Tropical Medicine, Nationalestraat 155, 2000 Antwerp, Belgium

**Keywords:** Cystic echinococcosis, Disease control, Dog, Sheep, Neglected zoonosis, Anthropology, Focus group discussion, Morocco

## Abstract

**Background:**

Cystic echinococcosis (CE), a neglected zoonosis caused by the larval stage of the tapeworm *Echinococcus granulosus*, remains a public health issue in many developing countries that practice extensive sheep breeding. Control of CE is difficult and requires a community-based integrated approach. We assessed the communities’ knowledge and perception of CE, its animal hosts, and its control in a CE endemic area of the High Atlas Mountains, Morocco.

**Methods:**

We conducted twenty focus group discussions (FGDs) stratified by gender with villagers, butchers and students in ten Berber villages that were purposefully selected for their CE prevalence.

**Results:**

This community considers CE to be a severe and relatively common disease in humans and animals but has a poor understanding of the parasite’s life cycle. Risk behaviour and disabling factors for disease control are mainly related to cultural practices in sheep breeding and home slaughtering, dog keeping, and offal disposal at home, as well as in slaughterhouses. Participants in our focus group discussions were supportive of control measures as management of canine populations, waste disposal, and monitoring of slaughterhouses.

**Conclusions:**

The uncontrolled stray dog population and dogs having access to offal (both at village dumps and slaughterhouses) suggest that authorities should be more closely involved in CE control. This study also highlights the need for improved knowledge about the transmission cycle of the parasite among communities and health professionals. Inter-sectoral collaboration between health staff, veterinarians, and social scientists appears to be crucial for sustainable control of this parasitic zoonosis.

**Electronic supplementary material:**

The online version of this article (10.1186/s12889-018-6372-y) contains supplementary material, which is available to authorized users.

## Background

Cystic echinococcosis (CE), also known as human hydatidosis, is a neglected zoonotic parasitic disease caused by the cestode *Echinococcus granulosus.* Dogs and wild Canidae are the definitive hosts, while domestic Ungulates act as intermediate hosts. Humans are accidental dead-end hosts. Considerable phenotypic and genetic variability exists within the *E. granulosus* species*.* Several strains have been identified with differences in intermediate host spectrum, biological characteristics, and geographical distribution [1]. Globally, most human cases of CE are caused by the sheep strain *E. granulosus* sensu stricto (characterized as the genotypes G1, G2 and G3) [2]. A common misconception is that humans become infected when eating uncooked meat. In reality, CE is transmitted via the faeco-oral route. Intermediate hosts become infected through ingestion of parasite eggs that are passed through the dog’s faeces. Humans are infected when consuming contaminated food or water, or through close contact with infected soil or objects (with subsequent ingestion of eggs), including dogs’ mouths and fur. An infection in livestock and humans leads to the development of a hydatid cyst in the liver, lungs, or more rarely, other organs [3]. While animals rarely show clinical symptoms, clinical signs and symptoms in humans may develop months or years after infection, and are caused by the expanding cysts and inflammatory reactions [4]. Severe cases can only be treated surgically.

CE is a worldwide health problem primarily affecting pastoral and poor rural communities where people raise livestock in close contact with dogs fed on raw offal [5]. The latest global burden of CE is estimated at 184,000 Disability-Adjusted Life Years (DALYs), − one DALY being defined as 1 year of healthy life lost due to ill-health, disability and/or early death -, for a total cost of US$3 billion attributed to CE-related health care costs and losses in the livestock sector [6].

CE is highly endemic in North Africa and the Middle East. In these regions, incidence rates in humans can exceed 50 per 100,000 person-years [7, 8]. In 2012, Morocco recorded an average of 5.2 surgical cases per 100,000 inhabitants (DELM, 2012) and a mortality rate estimated at 2–3% [9]. A retrospective survey (2007–2017) in the province of Khénifra shows a maintained incidence rate of 8.62 per 100,000 inhabitants (Ezzahra F et al., unpublished data). The last epidemiological study in the Mid Atlas showed a prevalence of abdominal CE of 1.9% based on ultrasound examination [10]. Official data from the Ministry of Health reported 722 surgical CE cases in 2014 [11]. The overall annual cost to both the Moroccan health and livestock sector has been valued at nearly €1,000,000, though data are fragmented [12]. According to a study conducted in five regions of Morocco, prevalence of infection ranges from 23.0% in cattle, 17.8% in Equidae, 12.0% in camels, 10.6% in sheep, to 1.9% in goats. In rural areas, one dog in two carries *E. granulosus* [13]. A study conducted in the province of Sidi Kacem (northwest Morocco) between 2010 and 2011 found an *E. granulosus* infection prevalence of 35.3% in pet dogs [14]. Although Morocco is considered a highly endemic country for CE [7], this obvious lack of data illustrates clearly the persisting neglect of zoonotic diseases in terms of funding opportunities for epidemiological research, of national health priorities, and consequently of validation of effective CE control strategies and of efficient surveillance measures.

Illiteracy, weak infrastructure and limited economic resources in rural and suburban areas in Morocco play a major role in the distribution of CE. The irrigation systems, extensive sheep breeding, high numbers of stray dogs, and poor hygiene in slaughterhouses create ideal conditions for transmission [15]. Both women involved in agricultural activities, and children who come into close contact with dogs, are particularly at risk [16]. Control of echinococcosis is difficult due to the complex nature of the parasite’s life cycle, the number of animal species acting as potential (intermediate) hosts, and the requirement for intersectoral cooperation between agricultural, veterinary, and health authorities [17]. Nowadays, the Eg95 vaccine for sheep [18], registered in some parts of the world, combined with cestodicidal treatment of dogs, shows a strong potential to control this disease [19].

Although the Moroccan Government established an “Inter-Ministerial Committee” in 2007 and drew up guidelines to combat CE [20], the disease remains a significant public health problem in this country. Currently, hydatidosis control program managers in Morocco wish to gain a better understanding of the type of risk behaviours that persist in rural populations, and why they persist. This may improve the impact of their control efforts. Dar and Alkarmi (1997) were the first to emphasize the importance of socio-economic and cultural issues in CE control in the Maghreb and the Middle-East [21–25]. Several authors have highlighted the role of human behaviour in the epidemiology of (re-)emerging parasitic zoonoses. Studies in Asian [26–28], South American [29, 30], and sub-Saharan African populations [31], as well as the Mediterranean region (primarily Tunisia and Algeria) [32–35], indicate that human risk behaviour associated with poor understanding of the parasite life cycle by the population has a negative impact. However, no study has extensively studied the socio-cultural determinants influencing human behaviour and CE transmission, and no research is available for the High Atlas region.

The objective of this study is therefore to document the reasons behind certain risk behaviours and the socio-cultural determinants hindering the control of CE. By assessing the knowledge gaps about the *E. granulosus* life cycle and transmission, the role of dogs in this rural society, the community perceptions, practices and knowledge regarding sheep and offal management, the study aims to propose more appropriate and effective strategies to overcome barriers, and hence contribute to better control of CE.

## Methods

### Study area

We conducted a qualitative research project based on focus group discussions in a predominantly rural area in the Northern fringes of the High Atlas Mountains, 30 to 50 km south of Marrakech in the Al Haouz province of Morocco. The study region was selected based on reports indicating high prevalence of *E. granulosus* in humans, sheep, and dogs (Report of the Haouz office, unpublished data), presence of extensive sheep breeding, a large number of free-roaming dogs, and backyard slaughtering without veterinary inspection. Six rural municipalities and one town (Tahannaout) were selected with the approval of the local authorities. The ten villages located among these seven municipalities were randomly selected from villages where the Caïd (chief of the rural municipality appointed by the state) agreed to the study.

The El Haouz province has a population of 484,312 (RGPH^1^ record from 2004), of which 90% is rural. The main ethnolinguistic group in this area is the Berbers. Their religion is mainly Muslim, and they speak the *Atlas Tamazight* language, which differs from Arabic.

Agricultural activities include extensive sheep breeding and growing of cereals and olives. The livestock population is about 135,265 heads divided into sheep (86%), goats (8.5%), Equidae (3%), and cattle (2.6%).

### Study design

Twenty focus group discussions (FGDs) were conducted in ten *douars* (villages), with a total of 175 participants (83 women and 92 men). In exchange for their time, participants were offered veterinary services. To respect the gender sensitivity of rural Muslim culture, ensure women’s participation, and maximize disclosure, none of the FGDs were mixed gender. Based on the initial study design, we held separate homogeneous FGDs with male (*n* = 8) and female (n = 8) villagers, and male butchers (*n* = 2) since these groups have supposing contrasting perspectives and exposure risks regarding health in general, sheep keeping, and the use of dogs. In the course of the field work we visited a high school where we conducted FGDs with female students (n = 2) initially selected under the same eligibility criteria of the other women groups. Heterogeneous groups are likely to hamper the quality of the data [36, 37]. The villagers were mostly sheep farmers, and the FGDs were conducted in Berber or Arabic languages, according to the audience. The number of FGDs conducted allowed us to reach “theoretical data saturation” [38].

### Data collection

Data collection took place from October to November 2009. The FGDs consisted of approximately 8 participants (Table 1), selected on the basis of their availability and willingness to participate. The question guide was pre-tested in one FGD for each gender among veterinary students from Rabat and fine-tuned during the course of the field work in the study area.

**Table 1:** Characteristics of the focus group discussions (FGD) by rural municipality

FGD No.	Municipality	Category	Number of participants	
			Female^a^	Male^a^
1	Tahannaout (town; 930 m^b^)	Students	9	
2		Students	6	
3	Aghouatim (510 m)	Villagers		7
4		Villagers	6	
5	Oukaimeden (2670 m)	Villagers	11	
6		Villagers	12	
7		Villagers	10	
8		Villagers		7
9		Villagers		12
10	Amizmiz (1100 m)	Villagers	11	
11		Villagers		6
12	Aghmat (670 m)	Villagers	7	
13		Villagers	7	
14		Butchers		8
15		Villagers		11
16	Ourika (860 m)	Villagers	4	
17		Villagers		9
18		Villagers		11
19	Aït Ourir (680 m)	Butchers		8
20		Villagers		13
			*N* = 83	*N* = 92

^a^Separate FGDs for women and men

^b^Meters Above Sea Level

To ensure quality of the data collection, two investigators (ST & HS) attended every discussion. Three trained facilitators, all familiar with Arabic and Berber languages, switched roles for each discussion. All FGDs took place in a quiet room in the village (e.g., a living room, classroom, meeting room in house of Caïd, medical consultation room, or administrative room in a slaughterhouse).

The discussions lasted about 40 min. The topics covered are presented in Table 2. The topics addressed were slightly adapted according to the group to ensure their interest and active involvement in the discussion (see Additional file 1).

**Table 2:** Discussion guide by topic and group

Topic	Topic addressed		
	Men^a^	Women^b^	Butchers
*1) Knowledge and perceptions of echinococcosis*	Y	Y	Y
• Knowledge • Treatment & prevention • Impact			
*2) Sheep management*	Y	N	N
• Role of sheep • Management problems • Cysts			
*3) Perception of dogs*	Y	Y	N
• Positive and negative aspects of dogs • Feeding of dogs • Stray dogs			
*4) Perception of control options*	Y	Y	Y
• Stop feeding dogs with sheep cysts • Feeding of dogs by owners • Burying or burning sheep carcass/offal to prevent dogs from eating them • Discourage dog ownership • Kill stray dogs • Replace sheep with goats			
*5) Slaughtering practices and cysts in sheep*	N	Y	Y
• Slaughtering practices • Waste management • Cysts			
*6) Hygiene*	N	Y	N
• Hand washing • Water use			
*7) Economical aspects*	N	N	Y
• Price setting • Knowledge • Treatment & prevention • Impact			

^a^Sheep farmers

^b^Farmers’ wives and female students

Legend: *Y* Yes, *N* No

All discussions commenced with questions on disease knowledge in order to ascertain whether participants spontaneously linked CE to any hosts (sheep and dogs), and whether other transmission factors would be suggested. All discussions were video-recorded except for one FGD with women who refused. Therefore, this FGD was audio recorded only. The facilitator was always assisted by a reporter.

### Data processing and analysis

All FGD recordings were transferred from the video-camera to the computer of ST and the files were burned to Compact Discs as back up and to be shared among the research team. TR and the two field assistants (Ahlam Marossi and Laila El Jirari) transcribed all recordings in Word documents and translated them from Arabic or Berber into French. To improve reliability, two researchers (ST & TR) independently reviewed the written transcripts before entering them into the analysis software, being aware that despite the precautions taken some original meanings might have been lost. Text analysis of the transcriptions and the notes taken during the FGDs was supported by the use of NVivo 10® software (QSR International Pty. Ltd., Melbourne, Australia, 2008), which allows data classification and sorting, and exploration of relationships and trends. Three investigators (ST, AM & TR) each independently coded the major themes emerged from each topic using an inductive approach. They discussed any differences until consensus was reached.

The main results of each topic listed in Table 2 are described in more detail and illustrated with anonymous quotes, chosen for their appropriateness and revealing quality. The order used to present opinions and ideas shared by the participants for each topic reflects the level of importance given by the participants to these topics (going from a strong to a weaker consensus). The purpose is to highlight observed patterns and respect what was said in the discussions as much as possible. In view of their substantial input, participants did not seem intimidated by the venues. In general, men were more talkative than women. The ranking of perceived acceptable or non-acceptable control measures included in Table 4 was obtained by creating new PROS and CONS categories and sub-categories for each measure based on the word of the participants and cross-cutting them.

### Ethical considerations

We obtained ethical clearance from the Agro-Veterinary Institute Hassan II Biomedical Research Ethics Committee (003–02-10). We sought approval from local authorities and community leaders before the study commenced. Finally, we sought individual oral consent from all FGD participants to video-record the discussion. Participation was entirely voluntary and no names were recorded. We took care to phrase the questions appropriately and respectfully.

## Results

### Life cycle of the parasite and knowledge gaps

This section is about participants’ knowledge and perceptions of CE in humans and sheep.

#### CE in humans

Most participants were aware of the disease, knew it mainly affects women and children, and were of the opinion it is now more widespread than in the past. Most of the CE cases they knew were not from their village but quite a few participants mentioned cases among family members or their neighbours. Five focus group participants (three women, a butcher, and a man) said they had a cyst removed surgically a few years earlier.

*“How many women and children infected? About animals, it must be 90%, especially in the area of the mountain where there are many dogs. It is widespread, and people do not know anything, children run alongside, anyone can be infected by the cyst to the liver or other. People are not aware, they know there are diseases, that it has consequences but do not know that dogs are the cause, there is a letting go. The disease we know, they say that the cause is dogs, but there is negligence from all around”.* (Focus group\Women Ourika municipality)

In Arabic, “*Alakyass almaiya*” means hydatid cysts (in human and sheep). Some names given by the participants to this disease were more closely linked to cysts (*cyst disease*, *livestock cysts, hydatid cysts disease*), others to dogs (*dog disease*, *cyst of dog*, *dog microbe, the dog trouble*) and some also to the liver (*liver disease*, *cyst of the liver*). No specific local name was given to CE in humans, except for the term “*Tamought*”, which in Berber means “disease”. Respondents identified the cause of CE as a “*microbe*” or a “*virus.*” In all the discussions the term “*parasite*” was only mentioned once by the participants.

The women mentioned the liver as the main organ where cysts were located. They also listed the uterus (vagina, ovaries), belly (intestines, kidneys), and lungs (only twice).

Hydatid cysts were perceived as a dangerous, deadly and evil disease, painful also (especially by women), and affecting health. Few stressed the importance of early treatment but mentioned that this is costly. Only one woman considered this disease not to be contagious.

Very few were able to discuss or describe the symptoms, and those who could describe them had personally experienced CE. They mainly mentioned loss of energy (difficulty to move, sleep, or eat), digestive disorders (swollen belly, vomiting), loss of weight and becoming pale-yellow. Two women said that these cysts also prevented women from getting pregnant.

Although most participants said that surgery and medication were the main treatment for this disease (even if these were not perceived as effective), they also mentioned that those infected rarely go to hospital as a first recourse. They would first seek healing from traditional healers. As a result, CE diagnosis and treatment is often delayed. Sometimes misdiagnosis contributes to this delay.

*“It is the liver jaundice. You take olive oil but without any result. A woman told me to take honey and sulfur, but I have been told that it is not good, you could die (laughs). I looked for honey, but my mother did not let me eat it, she told me it is dangerous. So, I went to see the doctor. Once the X-ray was made, he asked me if we had dogs in the village. I said yes. He told me that dogs cause this cyst, that I ate the (sheep) meat or even the hair of the dog that I have inhaled without noticing it. (…)”.* (Focus group\Women Amizmiz municipality)

Early surgical removal of the cyst(s) was considered to provide a complete cure, though a few women stated that cysts might recur. Students considered surgery to be risky.

Men especially identified dogs most often as the cause of CE in people, followed by consumption of infected offal (liver) or infected meat. Eating or drinking ware touched by a dog, touching or ingesting their hairs, contracting a bite, or contact with their breath, saliva, or dog in labour were mentioned as additional modes of transmission (see Additional file 2a).

Poor hygiene, cats, animals in general, infected livestock, climate (cold, wind, humid, polluted air), and contaminated drinking water were mentioned as other potential sources of infection. For many others, the origin was either unknown, or clearly not the consequence of eating mutton (see Additional file 2b).

In contrast to male villagers, the butchers saw infected offal rather than dogs as a cause of CE. They believed that even being rich or having good personal hygiene did not prevent anyone from getting a cyst. Compared to men, women pointed less to dogs or offal as the cause of cysts in humans (see Additional file 2c).

#### CE in sheep

While the topic “hygiene” was specifically discussed with women, the “sheep management” one was specifically discussed with the male villager groups. Therefore, answers from men dominate this section. However, this does not mean that men were more knowledgeable about this issue than the three other groups.

Cysts were observed in sheep by men at varying frequencies, and most commonly during the feast of *Eïd el-Kebir* when they slaughter sheep at home to celebrate the Sacrifice of Ibrahim. Among butchers, only one person reported that he also often observed cysts. Other infected animals mentioned included bovines, goats, and rabbits.

“*If you want, I can give you an estimate. For example, let’s say I kill 10 or 15 sheep, I would say that I found cysts in 5 or 6 of them, and me, I slaughter not often, just for the Eïd or other occasions, such as for example during the harvest. We find the cysts during the harvest period. My father used to slaughter a sheep or a goat whenever we started the wheat harvest, and at that time exactly the animals showed cysts.*” (Focus group\Men Oukaimeden municipality)

Cysts were associated with sheep living in close contact with dogs, sick sheep, white sheep, sheep from a specific *douar* or from the mountainous region, sheep grazing along the *oued* (river), or on meadows. Cysts were more often observed during the dry season or the harvest.

All groups concurred that cysts were mostly observed in the liver of slaughtered sheep. The lungs and intestines were also mentioned. Three butchers specifically mentioned that cysts were also known to be found in gallbladder, kidneys, and throat.

Most participants did not know the origin of the infection. The dog was mentioned most often as the cause of cysts (dogs eating infected offal, living together with animals, dog smell/ bite), followed by water (rain, river, hot water), infected offal, different causes according to the animal (butchers), and finally sheep feed (especially salt) (students) (see Additional file 2d).

According to one butcher, humans were also a source of infection for sheep. None of the participants reported observing clinical symptoms of CE in live sheep, although finding cysts at slaughter was often associated with lower weight gain during the fattening period.

Consumption of the cysts was almost unanimously considered as a threat to human health, though not always related to CE. Eating infected offal was considered harmful for dog and sheep health (mentioned by the butchers and the women). One group of men stressed that cooking alone did not kill all the germs. One man referred to it as the “evil eye.” The few participants who did not perceive the cysts as dangerous explained that they did not consider them to be a disease (e.g. some people considered the cysts as God’s creatures living inside the animals and did not believe there was anything bad about them), cooking could kill the cysts, though they had a bitter taste and disgusting aspect. Quite a few respondents mentioned that they could not properly evaluate the danger of these hydatid cysts in general.

Finally, we found that there was some confusion during the discussion between hydatid cysts in the human liver and gynaecological cysts. Participants also made some distinction between the type of cysts found in animal meat and that some aspects of rabies transmission were included into the perceived life cycle of *E. granulosus,* e.g. dog bites as a mode of infection.

### Dogs: perception, role, and status

Overall, dogs were perceived as harmful animals, especially stray dogs, which were seen as useless. Men had the most negative perception about dogs, female students the least negative. Thirty-nine topics were mentioned regarding the dogs’ harmfulness. We classified them into seven more generic emerging themes relating to: hygiene (I), nature of the dog (II), roaming pattern (III), care (IV), human health (V), general nuisance (VI), and safety (VII). Themes and their respective sub-themes were listed from most to least mentioned (Table 3).

**Table 3:**
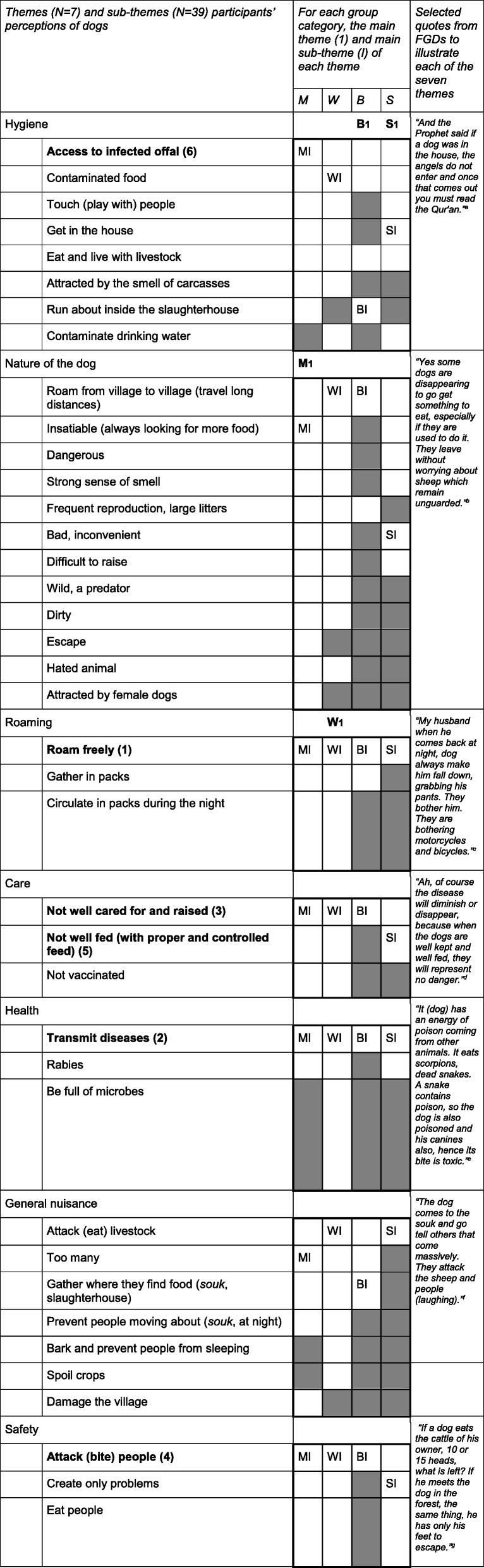
Aspects perceived as negative for dogs, and for each of the four categories in the FGD

**(1)** to **(6)**: The six main nuisances mentioned regarding dogs

*M* Men, *W* Women, *B* Butchers, *S* Students

MI: most mentioned sub-theme by Men; WI: most mentioned sub-theme by Women; BI: most mentioned sub-theme by Butchers; SI: most mentioned sub-theme by Students

**M1**: most mentioned theme by Men (Dogs’ nature); **W1**: most mentioned theme the by Women (Moving); **B1**: most mentioned theme by Butchers (Hygiene); **S1**: most mentioned theme by Students (Hygiene)

: No quotes found to be coded in this sub-theme

^a^Quote from female students focus group from Tahannaout municipality

^b^Quote from male focus group from Aghouatim municipality

^c^Quote from female focus group from Aghmat municipality

^d^Quote from male focus group from Amizmiz municipality

^e^Quote from female focus group from Aghmat municipality

^f^Quote from butchers focus group from Aghmat municipality

^g^Quote from female focus group from Aghouatim municipality

Most topics covered under negative aspects of dogs related to free-roaming owned dogs as well as stray dogs, which were perceived as a threat to people’s health and security. This is in contrast to non-roaming owned dogs, which have a role in the community such as guarding, shepherding and hunting. In addition, owned dogs are usually better taken care of, though from a hygiene point of view, they were still very much perceived as a threat.

“*… But the most dangerous category are dogs that do not serve to guard, herd or hunt. People feed them occasionally, but they stay and sleep outside next to the houses. The souk day, they go and hang out near the slaughterhouse, and other days, they fetch carcasses of dead animals in the ravines. Then they go in the forest where they breed and multiply and become a real problem…*” (Focus group\Men Aghouatim municipality)

Some respondents compared dogs to wolves (or to wild animals, predators), gathering in packs and preventing people from moving about freely, especially at night or around places where dogs can find food. Five different groups even referred to the same incident related to feral dogs that had attacked and eaten a teacher (see Additional file 2e).

Female students were more likely to view dogs as pets than other groups were. They agreed that not taking good care of these animals, and the associated hygiene issues were a problem, especially if the dogs entered the house. For the butchers, the hygiene issue also came first (in Morocco, dogs are prohibited in slaughterhouses), but the care and the safety risks posed by dogs were not perceived to be that much of a problem. Female respondents especially stressed the risks of being attacked or bitten by dogs. Men perceived the nature of dogs as the main negative aspect and in particular mentioned their insatiability.

A few positive characteristics of dogs were cited such as their resistance/strength, mainly as a result of their ability to find food by themselves. Some participants, especially among the student groups, remarked that dogs also have a soul and therefore the right to live. For some, dogs were companions and essential in their life. None of the butchers had anything positive to say about dogs. Some of the men used the term “*Hachakoum”* immediately after pronouncing the word “dog”. *Hachakoum* means “excuse my language” and is used after an obscene, unhealthy, dirty, noxious word. In Morocco, rural populations use this term to designate dogs, donkeys, pigs, rubbish, faeces, etc. Some participants talked of the obligation to wash anything touched or contaminated by a dog seven times to purify the place and allow the angel to return. It compels owners to take good care of their dogs (see Additional file 2f).

### Sheep management and slaughtering practices

Sheep were perceived as “life pillars”, a source of income and savings, as well as carrying prestige, especially during the religious festival of *Eïd el-Kebir*. Participants saw many advantages of sheep compared to goats, stating their economic and cultural importance. Women take care of the livestock (feeding, watering, cleaning the sheep pen), and this was also acknowledged by a few men (see Additional file 2g).

Sheep do not cause any nuisance or pose any threat to human health with the exception of the hydatid cysts or an infected liver. Some respondents said that imported sheep from another Moroccan region tend to get sick more easily.

Men said they were in charge of slaughtering and selling the meat at the *souk* (market). Slaughtering is mainly carried out at home, unless by butchers, and primarily for the *Eïd el-Kebir festival*. Other occasions such as weddings and harvest festivals were also mentioned. Animals are killed inside the house, in front of the house, in the stable, at the *souk*, on the ground, or on a tree trunk, and usually not far from a water source.

While the men’s role (sheep owner, father, family chief, grandfather, imam) is to slaughter the sheep, the women’s responsibilities are to wash, manage offal, and prepare the meat and tea.

“*She washes the stomach and prepares the liver and cooks it, and prepares food for the children (…) And it is also she who deals with waste disposal and cleaning.*” (Focus group\Men Ourika municipality)

Sheep were also clandestinely slaughtered by butchers or in official slaughterhouses. Besides slaughtering, the butchers’ roles is weighing/selling meat, and inspecting organs. There are different categories of butchers, such as *Maalem* (master butcher) and his trainee, *skaytiya* (retailer, wholesaler), and *aarif* (butcher spokesperson). The Imam was also mentioned as being present from time to time to say a prayer before the throat of the animal is slit (see Additional file 2h).

Meat inspection is compulsory, and performed by veterinarians who sanction clandestine slaughtering. Veterinarians inspect the lungs, liver, head, and foetus, while butchers only look at liver and lungs. The livestock owners said they examine the liver, lungs, heart, and gall-bladder when carrying out a home slaughtering. A few men also mentioned corruption, lack of veterinarians, as well as poor slaughtering conditions (lack of hygiene) (see Additional file 2i).

Intestines, liver, lungs, stomach (and its contents), gall bladder, heart, and foetus are examined after slaughter. Bones, horns, and gall bladder are discarded, as well as the stomach contents, which are sometimes used as fertilizer. Offal, when considered healthy, as well as heads and legs, are mainly kept for the preparation of traditional dishes. The butchers consider offal as real sellable meat, and lungs, heart, intestines, and especially the liver are noble organs with cultural and economic value. It is considered a great disappointment, frustration, and shame especially for the head of the household who bought or raised the sheep, if the animal slaughtered for the *Eïd el-Kebir* celebration did not have a big, healthy liver.

“*Besides, you know what? People here are poor, and somebody who slaughters a sheep from time to time will not easily throw the liver away that he was looking forward to, to prepare a nice barbecue for him and his children. And there are also those who slaughter a sheep only once a year, on the day of the Eïd Elkebir, you understand? Sometimes people see that there is some stuff in the liver and close their eyes or give no importance because their children wait to eat this liver.*” (Focus group\Men Amizmiz municipality)

The reasons mentioned for discarding offal -besides the presence of hydatid cysts- were the fact that they were inedible (e.g. stomach contents), unhealthy, not useful, not appetizing, or presenting suspicious lesions (abscesses, white spots, nodules, blackening, holes). While these were mostly discarded preventively, the repulsive aspect of their consumption was a major motivating factor as well.

The management of offal with hydatid cysts can approached three different ways, independent from location/type of slaughtering (see Additional file 2j): (1) removal of the infected part of the organ only (the main method and only method performed by the butchers), (2) removal of the entire organ, and (3) consumption of the whole offal. The latter approach was the least mentioned and only by a few men and women.

Different treatments of the infected parts were explained by the participants. Most treatments enabled dogs access to the cysts, including frequently throwing the offal with cysts to the dogs, or ‘discarding the infected parts’ without clear indication of where and how they were thrown away. Places where the infected organs were disposed of included: garbage pits close to as well as far away from the house, in a plastic bag, in the river, in the street, and adjacent to the slaughterhouse. Practices preventing the dogs from accessing the cysts included burying (which was not a habit in the region), burning, drying, and staining with *Cresyl,* a chemical disinfectant based on phenols which gives a repugnant smell to the condemned meat. There was a consensus among the butchers that dogs should not be allowed in the slaughterhouse, although one woman said: “w*hen the butchers come out, the dogs are eating*” (Focus group 10\Women Amizmiz municipality), which means that dogs do have access to waste inside the slaughterhouse.

In most cases, the aforementioned actions did not prevent dogs from accessing the infected offal. With the exception of the butchers, it is important to highlight that more participants mentioned behaviours which allow dogs access to infected offal, rather than those behaviours preventing it. This gap was more pronounced among men. Explicitly giving infected tissues to dogs (and cats) was the action most often mentioned, and mainly by men.

### Control measures: pros and cons

In this section we present the participants’ knowledge and perceptions of several suggested CE control measures (Table 4).

**Table 4:** Participants’ knowledge and perceptions of suggested CE control measures: pros and cons

Rank	Perceived acceptable control measures		Perceived non-acceptable control measures	
	Measure	Advantages	Measure	Obstacles
1	Kill stray dogs only	Help eliminate dogs and their threats	Replace sheep with goats	Against the culture (food habits, religion); many disadvantages of goat herding
2	Stop feeding dogs with sheep cysts	Contribute to decreasing the disease	Kill all dogs	Some dogs have a role (useful) and the right to live (have a soul)
3	Feeding dogs personally	Prevent dogs going out to look for their food and returning with diseases	Stop feeding dogs with sheep cysts	Cysts can be found anywhere else (*souk*, slaughterhouses)
4	Prevention versus treatment^a^	More efficient than treatment	Feeding dogs yourself	Impossible to educate a dog (big appetite)
5	Kill all dogs	Fewer problems	Kill stray dogs only	Dogs always reappear (reproduction difficult to control) especially if culling campaigns are not carried out regularly
6	Bury infected offal	Prevent dogs’ access to offal	No more owning dogs	Dogs are needed
7	Stop owning dogs	Not specified	Do not throw away carcases	Too costly and time consuming to bury them
8	Stop throwing away carcasses	Avoid the bad smell	Bury wasted offal	Dogs have a strong sense of smell and offal is not buried deep enough
9	Replace sheep with goats	Healthier meat with fewer cysts found (less contacts with dogs)	Burn infected offal	Too costly
10	Burn infected offal	Prevent dogs having access to offal and people having to retrieve them Avoid bad smell	Reduce dogs’ access to slaughterhouses^b^	Not the best method to control the disease because it does not come from the slaughterhouse but from the pastures

^a^Control measures suggested only to men, women and student groups

^b^Control measure suggested only to the butchers

Both men and women’s groups discussed the positive views and obstacles relating to implementation of CE control measures. In general, female students were more often opposed to these control measures, especially to those related to dogs. Butchers were generally more in favour of all these measures.

Of the ten proposed control measures suggested for discussion in the FGDs (Table 4), killing stray dogs was the one that got the biggest consensus from all the groups. However, a few obstacles mentioned included difficulty in controlling dogs’ reproduction, and the inefficiency of this action if it was not implemented regularly and long-term. The female students were the only ones who did not support the killing of all dogs. Both the women’s FDG and the female students highlighted the disadvantages of this measure, including the useful roles for dogs, the fact that dogs are living creatures and had souls (this also applies to stray dogs), and the need for management of dog carcasses. Some butchers feared this measure would never become properly implemented and would therefore not be sufficient to eradicate the disease.

Men were slightly more likely to be in favour of the prevention of feeding cysts to dogs than the other groups, and generally agreed that it could contribute to a reduction of the disease, but not to its eradication (see Additional file 2k). Nevertheless, men also said this was a bad solution as dogs would continue to find cysts elsewhere (*souk*, slaughterhouse, …); free roaming stray dogs could bring the “badness” from another source, and the implementation would fail due to lack of sensitization and people’s carelessness. In addition, few women across the FGDs considered this a bad strategy.

On the other hand, men generally considered burying offal an important measure, primarily to prevent dogs’ access to it. Similarly, it was primarily the men who were in favour of the idea of stopping the indiscriminate disposal of carcasses. The reasons for this are firstly to avoid the smell, secondly to implement the law, and thirdly, to prevent dogs’ or wild animals’ getting hold of the carcasses. Some men and butchers expressed scepticism about these measures for the following reasons: the high cost of burying or burning cadavers, the time required to do so, disposal of carcasses is not commonplace (lack of sensitization), and carcasses are not usually buried deep enough, allowing dogs to dig them up.

All groups opposed the replacement of sheep with goats, and men generally did not look favourably on feeding dogs (to prevent them looking for other food). According to the men, most local dogs are strays without an owner and are therefore uncontrollable. Dogs, in general, would always be on the lookout for more food, and dog food was too expensive.

Preventing dogs from roaming around in the slaughterhouse was only proposed as a control measure to the butchers who said that not only was this measure already implemented, it was also not the most efficient way of controlling the disease.

“*Men 8 (M8): But the disease is not here that it appears or in the slaughterhouse, but elsewhere when herds are circulating in the prairies and between villages.”*

*“M6: (When herds are circulating) with the dogs, two or three dogs.”*

*“M2: Some owners have four dogs, and sheep are not vaccinated nor dewormed. There is a misconception. He thinks that if he treats these animals they will not be in good condition anymore, and he maintains the presence of four-five dogs with him.”*

*“M6: And it is from the dogs that comes this disease.*” (Focus group\Butchers Aït Ourir municipality)

#### Additional measures suggested by the participants

In addition to these 10 strategies to control CE, participants also suggested alternatives for themselves and authorities (veterinary services and the government). They also asked to combine all the suggested control measures and include an improved sensitization.

##### Individual measures

Individual measures to be enacted by all included improved hygiene, especially before cooking (washing hands, food, and cooking utensils), blocking animals’ access to the house, avoiding consumption of food touched by dogs, avoiding consumption of hydatid cysts, and fencing pastures. Participants also suggested burying animal carcasses sufficiently deep and with the help of others.

Dog owners should take greater care of their animals, stop them roaming freely, feed and train them, and prevent their access to livestock when they are sleeping and being fed in their pen.

##### Measures to be implemented by the authorities

Control strategies suggested for implementation by veterinarians and the government mainly by male participants included dog vaccination (which does not currently exist for CE).

“*Women 8 (W8): But why Europeans live with dogs but are not affected by the disease?”*

*“W1: They vaccinate them.*” (Focus group\Women Amizmiz municipality)

A number of measures actually related to improved implementation of the aforementioned strategies included: creation of specific places for stray dogs, a more regular and synchronized culling of dogs, organized pick up of dog cadavers and infected offal, and more regular inspection of all carcasses by veterinarians. The latter should also provide more assistance to farmers (treatment and advice, even via a free phone number), encouraging farmers/people to take better care of their animals.

A male FGD advocated for the prohibition of slaughtering at home as a solution for CE control. They also suggested improved strategy implementation by imposing fines on those that do not apply the control measures, and the signing of implementation agreements.

##### Sensitization measures

Those who had heard of CE or hydatid cysts said they had been informed by the medical sector, the media, their network, veterinarians, charitable associations, religious organizations, schools, ministries, and butchers. Women received health information mainly from the media, and butchers only via veterinarians. Students obtained information from various sources, but not via veterinarians or butchers. Men learned about this disease primarily via the medical sector and associations (see Additional file 2l, m and n).

In some of the discussions there was a demand for raised awareness and health promotion for CE, at both village and individual level, but especially amongst dog owners.

Some specific recommendations were made for improved sensitization tools. Television, often used as a dissemination channel, was not considered efficient because villagers either did not have access to a television, or they were working in the field all day. In addition, explanations given in newspapers were often too difficult to understand and led to more confusion and misunderstanding. Hence, it was suggested to firstly make visits (face-to-face sessions) and tour the whole region, specifically rural areas which receive less attention than urban areas. Secondly, the display of illustrated posters in the Berber language was also deemed important because not everybody speaks or understands French or Arabic. In particular, women emphasized that everyone should share their knowledge about the disease with their family, friends, and community, and promote the adoption of appropriate behaviours. The health promotion messages and visits had to be carried out by “intellectuals”, and the scientific community. Media (mainly radio, and following face-to-face sessions), rural associations, health facilities, ministry of health, schools, religious representatives (imam), and veterinarians were suggested as appropriate relays for the dissemination of these health messages.

## Discussion

This qualitative study offers a deeper understanding of why rural communities in Morocco are engaged in behaviours resulting in the improper disposal of viscera at slaughtering, leading to high risk of *E. granulosus* infection in dogs [12]. The study was conducted in an epidemiological context representative of other rural settings in Morocco [10] and neighbouring countries [39], where CE is principally maintained in a domestic dog-sheep cycle and affects mainly women and children. Respondents described specific risk behaviours leading to a very high environmental contamination with parasite eggs including inadequate sanitary practices regarding offal management, abundance and proximity of free roaming dogs looking for food, and low awareness of transmission risks.

Similar to previous studies carried out amongst the Berber people from the Middle Atlas (central Morocco) [25], our results show that the occurrence of CE in both humans and animals is widely known in the studied community. Hydatid cysts and lesions were recognized and well described by our respondents, but their knowledge of the transmission cycle and the link to human disease was relatively poor and fragmented. In contrast to pastoralists [17] and findings from KAP study conducted in the Northwest of Morocco [10], our study population was well-aware of dogs being a source of infection, to the point of naming CE “dog disease” or “dog cysts”. However, how dogs get infected and how they transmit the disease to humans and livestock was not well known by many of the participants.

**Fig. 1:**
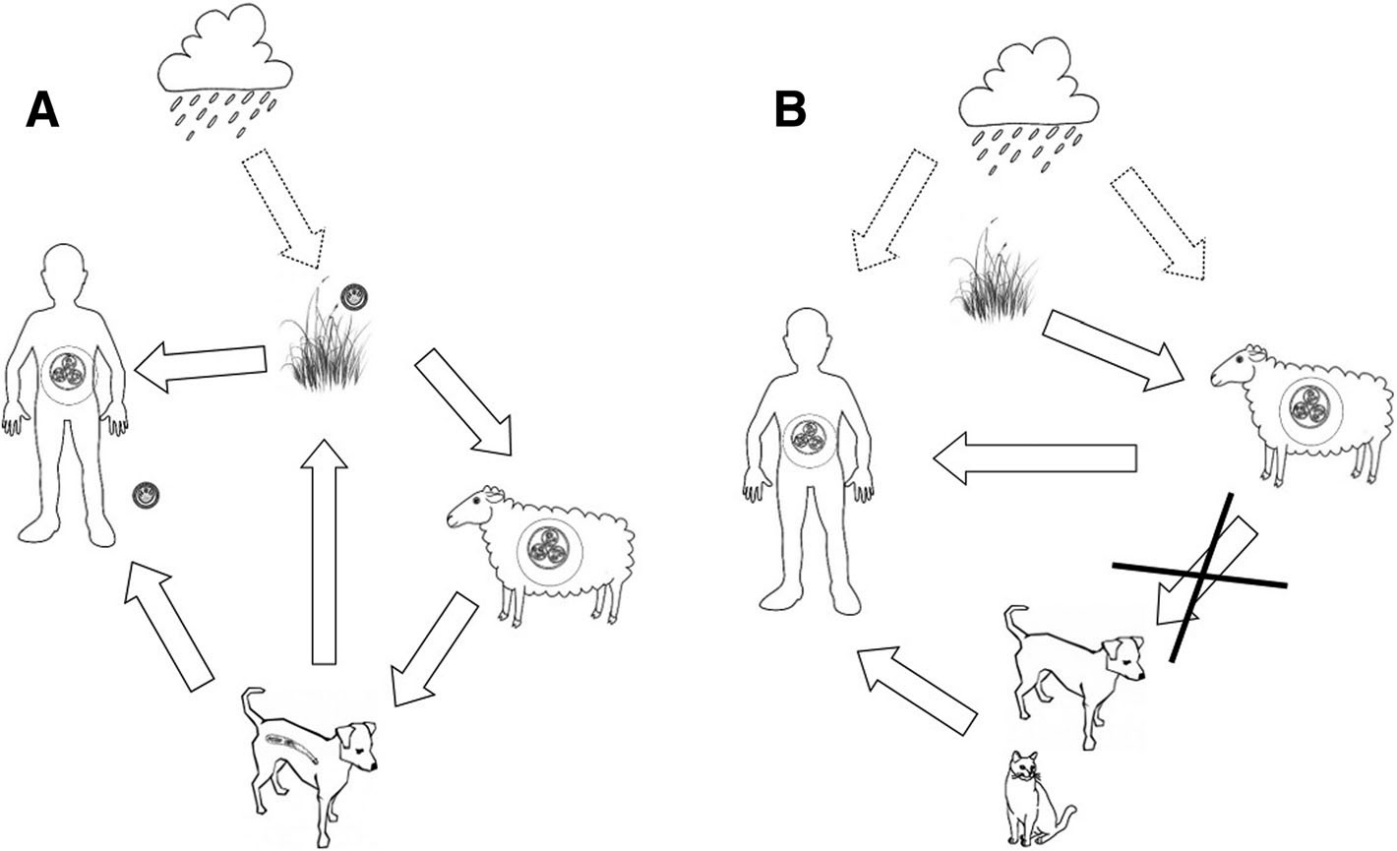
The real (**a**) and perceived (**b**) life cycle of *Echinococcus granulosus* in High Atlas, Morocco. Adapted from Marcotty et al. (2013). **a** Life cycle of *Echinococcus granulosus* in the High Atlas, Morocco. **b** Transmission routes as perceived by the Moroccan communities in the High Atlas (focus group discussions). **a** Adult *E. granulosus* live in the intestine of dogs and disseminate their eggs in the environment with the dogs’ faeces. Rains contribute to the egg dissemination, and the cold and humid climate allows their survival in the environment. Through direct or indirect contacts with dogs, ruminants (through grazing) and humans (through contamination of hands, food, and water) are infected by dogs’ faeces. Dogs become infected when feeding on organs infected with hydatid cysts (larval stage). **b** Dogs first, and infected sheep meat, a close second, are seen as the two main sources of human hydatidosis. Sheep, and to a lesser extent, humans, get infected in humid or cold climatic conditions or in unhygienic environments. Dogs, as well as cats, are thought to contribute to hydatidosis and other diseases in humans through saliva, hairs, and bites. Infection of dogs through feeding on infected organs is not perceived as a risk. (*The plain arrows represent the direct cause of disease and the dotted arrows represent the influence in the causal chain of transmission.*)

By partitioning the life cycle of *E. granulosus* according to its transmission paths between the three hosts (humans, sheep, dogs), and organizing all related quotes accordingly, we could picture participants’ perceived life cycle of the parasite (Fig. 1), and identify the corresponding knowledge gap(s). A perceived life cycle was described by Marcotty et al. (2013) using preliminary data [15].

As in our previous study on *Taenia solium* [40], very few people had a comprehensive view of the parasite life cycle. While some pieces of the puzzle were easily identified (e.g. risks dog poses to human health), others were often lacking (e.g. infection of dogs through feeding on infected organs), and some putative hosts were erroneously added (e.g. cats). Finally, some transmission paths between hosts were easily identified (e.g. humans getting cysts from dogs), although the knowledge on how the disease was transmitted from one host to the other was not always accurate (e.g. humans getting cysts from dogs by their breath).

In contrast with the recent Moroccan KAP study [10], we observed that the main knowledge gaps varied between the stakeholders. The female students had very poor knowledge in general. They perceived the threats from the dogs as less serious compared to the other groups, and saw dogs more as pets. Very few butchers identified the dogs as a source of infection for humans, as for them the main risk for contracting CE in humans was eating infected offal. On the other hand, butchers were well aware that dogs could infect sheep. Very few women but most men made the link between infected offal and dog infection. Yet, more women than men considered eating infected offal less of a health issue. For men, the biggest problem was the disease transmission from dogs to humans.

These data are very important to build efficient and adapted health promotion messages because the specific knowledge gaps explain several of the risk behaviours, especially regarding infected offal management. If women’s responsibilities are to clean and manage viscera but, at the same time, these women do not perceive the hazard of feeding dogs with infected offal, the parasite’s life cycle will be maintained. This finding is in line with other Moroccan studies that highlighted that safe disposal of offal with cysts is downplayed because only human consumption of cysts is considered holding a disease risk and not consumption by dogs [10, 41]. Undeniably, offal are very often made accessible to dogs, voluntarily or involuntarily, at slaughterhouses, *souks*, open air uncontrolled dumping, streets, and rivers, which is the main reason for the persistence of the disease [42]. These offal management practices however, are not only mediated by lack of awareness, but also by various social determinants including religion, gender, and food preparation norms of sheep offal, which influence how households consume and discard infected offal. During the *Eïd el-Kebir* festival, the Moroccan tradition consists of preparing organ meat such as sheep liver and heart on the day of slaughter. The liver is grilled first by the men of the family partly for practical and sanitary reasons (organs are best eaten fresh), but mainly because of the liver’s symbolic and social value which strongly competes with its commercial value [43]. As a “concentrated life form”, the liver of the animal is considered as the seat of the filial love, hence the importance of its physical integrity especially during the yearly sheep sacrifice festival. Other viscera are also seen as preserving the honour of the family [44] and attracting the “*Baraka*” (flow of blessings and grace) to the whole family [43]. Consequently, all damages, black spots, and cysts should be removed from the organs and disposed of, which often makes them accessible to dogs (see above offal management practices), allowing the maintenance of the parasite’s life cycle.

The place given to dogs by the Muslim culture and some related religious precepts translated from the Koran by Imams in *hadiths* (Muslim laws) explain why dogs should be well treated like all other God creatures (dogs have a soul and therefore the right to live) or killed if they attack, but also why they are so often blamed as the source of cysts found in humans even though they are asymptomatic for *E. granulosus* infection. A close relationship with them is discouraged since they are hygienically and spiritually perceived as impure animals. This is even more the case for “unwanted” stray dogs, because they chase away protective angels from entering a house. The dirt brought by impure animals needs to be washed away. These Muslim cultural and religious beliefs, referring to the miasma theory [45], constitute probably an underlying explanation of several practices regarding dog keeping in the Middle East and North Africa, where stray dogs are abundant, and where there is often no legislation in relation to responsible dog ownership and formal control of reproduction [46]. According to our results, dogs, even owned, most often roam freely, and this may be because tying them up would imply to physically touch their “impurity”. In the context of Tibetan herdsmen, Heath et al. [47] advocated for more participatory planning between dog-owners and community leaders to enable a choice of possible control strategies that suit both the social and economic status of a particular target community. However, waste management and hygiene negligence, both from the rural communities and the authorities, was often mentioned in our study as a barrier to any control measures. This discrimination of rural and more remote areas by the state and by the authorities felt by our studied population was also identified in recent studies conducted in Morocco [41, 48, 49].

Aside from the control measures discussed, health promotion, including health education and awareness creation, was spontaneously raised by some participants. Others recommended several methods for organizing and implementing these awareness-raising campaigns in a more effective way, e.g. face-to-face meetings, radio messages in Berber language, etc. These proposals should certainly be considered when designing sensitization programs.

The dissemination of the life cycle of *E. granulosus* and risk factors contributing to human infections is the biggest challenge for CE control [47]. Ducrotoy et al. [49], who evaluated an integrated health messaging intervention for five zoonotic diseases in northwest Morocco, including CE, showed similar findings. Their intervention had different outcomes according to the target audience (men, women, and children) because of the educative role and norms at household level, and children remained the most receptive group [49]. Behavioural Change Education regarding dog keeping, livestock husbandry, personal hygiene, and home-based slaughter practices is important but would never be enough to tackle conceptual and operational challenges in the design of efficient interventions at the “human-animal-ecosystem” interface [41].

The scope of our research mainly focused on the biosocial dynamics of the parasite at the household level, yet the ones in place at slaughterhouse level also present a lot of challenges for controlling this disease, as highlighted by Bardosh et al. [41]. The butcher groups made fewer comments regarding dogs because the topic of perception, role, and status of dogs was not included in their FGDs (see Table 2). As a powerful lobby group and because of the complex socio-political processes that exist among the different stakeholders involved in slaughterhouses management such as veterinary technicians and politicians [41], butchers may have strategically controlled their answers when asked about the presence of dogs in the slaughterhouse, the management of discarded offal, and meat inspection to avoid getting attention from the authorities and the implementation of additional control measures on their work.

Sheep management was not included in the discussion guide for women. However, we discovered that women are the ones taking care of the livestock (although the decision-making still remains the responsibility of the men). This observation further emphasizes the importance of identifying, understanding, and integrating all the pertinent actors related to the targeted health problem when elaborating a disease control program, and thus also including the role of women in livestock management.

## Conclusions

The observed differences regarding CE perception between four social strata raise the challenge of engaging these different social groups in CE prevention and control. This means that looking at each group category and their prioritized control measures might be a good approach as previously advocated by Battelli (2009). However, to remain feasible and “salable” to program managers, the specificities of each group should be jointly prioritized through a consensual participative approach.

For example, free roaming dogs are key in the continued disease transmission in our studied ecosystem. Supporting dog-owners by, identifying their needs, dog sterilization, awareness campaigns, mobile vet care, free deworming, etc., is essential for effective CE control, but interventions for the management of stray dogs in this rural remote area must also be designed. In our study, killing unowned stray dogs perceived as harmful was identified as the most acceptable control measure, despite Islam not allowing the killing of any living beings. Therefore, stray dogs should be visually distinguished from free-roaming owned dogs, by collars, ear tags, etc.. Local authorities should also elaborate integrated strategies through the rabies control program created in 2000 by the ONSSA (Office National de Sécurité Sanitaire des Produits Alimentaires)^2^ to manage the size of dog populations. Also, to restore the trust of the population in their local authorities and ensure a successful and long-term hydatid disease control program, integrating “mediators” such as local associations and NGOs could be explored.

As culture is always evolving, adapted control measures must be implemented and negotiated with the population to be effective and sustainable. Therefore, we suggest a reflective bottom-up approach of progressive problem solving starting with a mapping of all the stakeholders involved in this control program, and a qualitative evaluation of their respective knowledge of this program and its activities, their role, their perception of its efficiency, and implementation. At the same time, it would be beneficial when redesigning and implementing the activities of the Moroccan CE control program to improve CE knowledge of both medical professionals and communities via health promotion techniques, taking into account the socio-cultural context of these groups. Medical doctors working in these remote areas could play an important role in knowledge transfer of CE control, while students who were perceived and perceived themselves in our study as “vanguards” of behaviour change within their immediate families and the wider community could also make a considerable contribution.

Finally, vaccination of sheep was not included in the discussions as this was not available as a control option at the time of our study (2009). However, the Institute of Agriculture and Veterinary Hassan II (Rabat), together with the support of Belgian universities^3^ is currently evaluating the efficacy and acceptability of the Eg95 vaccine for sheep.

## Additional files

Additional file 1: Focus Group Discussion guides (Men, Women and Butchers) in English. The guides elaborated for the Focus Group Discussions for each of the three group categories (Men, Women and Butchers), documented in the research protocol and translated from French to English for the purpose of this publication. (DOCX 24 kb)

Additional file 2: Illustrative quotes from the Focus Groups Discussions (a to n). Quotes (a to n) selected from the Focus Group Discussions conducted in the Hight Atlas in Morocco (October - November 2009) to illustrate the results. (DOCX 18 kb)

## Abbreviations

CE: Cystic echinococcosis
DALYs: Disability-Adjusted Life Years
DELM: Direction de l’Epidémiologie et de la Lutte contre les Maladies
FGD: Focus group discussion
IAV: Institut Agronomique et Vétérinaire
KAP: Knowledge/Attitude/Practices
M: Man
ONSSA: Office National de Sécurité Sanitaire des Produits Alimentaires
RGPH: Recensement Général de la Population et de l’Habitat
W: Woman

## Footnotes

1 Recensement Général de la Population et de l’Habitat

2
http://www.onssa.gov.ma/fr/sante-animale/programme-de-prophylaxie/rage-animale

3
http://www.ares-ac.be/fr/cooperation-au-developpement/pays-projets/projets-dans-le-monde/item/78-prd-renforcement-de-la-strategie-de-lutte-contre-l-echinococcose-zoonotique-au-maroc-aspects-veterinaires-economiques-et-sociologiques
