# Supplementary material for: Community perception and knowledge of cystic echinococcosis in the High Atlas Mountains, Morocco
Source: BMC Public Health. 2019 Jan 28;19:118. doi: 10.1186/s12889-018-6372-y (PMC6350308; doi:10.1186/s12889-018-6372-y)
Supplement: Supplementary file 1 — Focus Group Discussion guides (Men, Women and Butchers) in English. The guides elaborated for the Focus Group Discussions for each of the three group categories (Men, Women and Butchers), documented in the research protocol and translated from French to English for the purpose of this publication. (DOCX 24 kb) [file 12889_2018_6372_MOESM1_ESM.docx]

**Additional file 1: Focus Group Discussion guides (“Men”, “Women” and “Butchers”) in English**

1. Men

**Knowledge and perceptions of echinococcosis**

Have you ever heard about cysts in people (men, women, or children) ? What is the name given? Where in the body are these cysts located? Is it frequent in this village ?

How important is this disease in relation to your health? What is the impact on daily life?

What happens when somebody develops such a disease? How do you treat it ? How can you prevent it?

What is the origin of these cysts according to you ?

How can we interrupt the development of these cysts? How can we control the cysts? How do we remove the cysts?

**Sheep management**

*Sheep role*

What are according to you the positive aspects of sheep keeping ? For which reasons sheep are useful? What are the negative aspects of sheep keeping ? What are they dangerous for ?

*Issues of management*

What do you experience as major issues regarding sheep management ?

Where are the sheep slaughtered ? How many sheep approximatively are slaughtered in this locality ?

Regarding home slaughter: How is it done ? Do you have a special location where to do it? What kind of specific precautions do you take? Who is involved in the activities related to slaughter ? What do you keep/throw away ? What do you do with the waste ?

*Cysts (Echinoccocus)*

Have you ever found white large cysts on a carcass of a sheep ? On which parts?

How often ?

What did you do when you found these cysts ?

What is the origin of these cysts?

Do you find them also in other animals? How similar/different are those cysts compared to these found in sheep ?

How harmful can be these cysts ? For sheep health? For human health?

What would happen if dogs eat these cysts? What would happen if a person eat these cysts ?

**Perception of dogs**

*Positive and negative aspects of dogs*

According to you what are the positive aspects of dogs ? For which reasons dogs are useful or needed? What are the negative aspects of dogs? In which case they represent a danger or a nuisance ?

*Feeding of dogs*

What dogs are fed with ? Do feed them yourself? Where are they looking for their food?

*Stray dogs*

What do you think of stray dogs ? For what are they useful ? For what are they harmful ? How do they impact your village (beside rabies) ?

**Perception of control options**

Imagine that we propose several control measures against echinococcosis (or use local name). What would be the principals obstacles to implement them:

Method 1 : Stop feeding dogs with sheep cysts ?

Method 2 : Feeding of dogs by owners? And their puppies ?

Method 3 : Burying or burning sheep carcass/offal to prevent dogs from eating them ?

Method 4 : Discourage dog ownership ?

Method 5 : Kill stray dogs ?

Method 6 : Replace sheep with goats ?

1. Women

**Knowledge and perceptions of echinococcosis**

Have you ever heard about cysts in people (men, women, or children) ? What is the name given? Where in the body  are these cysts located? Is it frequent in this village ?

How important is this disease in relation to your health? What is the impact on daily life?

What happens when somebody develops such a disease? How do you treat it ? How can you prevent it?

What is the origin of these cysts according to you ?

How can we interrupt the development of these cysts? How can we control the cysts? How do we remove the cysts?

**Slaughtering practices and cysts in sheep**

*Slaughtering practices*

Regarding home slaughtering: How is it done ? Do you have a special location where to do it? What kind of specific precautions do you take? Who is involved in the activities related to slaughtering ?

*Waste management*

What do you keep/throw away ? What do you do with the waste ?

*Cysts (Echinococcus)*

Have you ever found white large cysts on a carcass of a sheep ? On which parts?

How often ?

What did you do when you found these cysts ?

What is the origin of these cysts?

Do you find them also in other animals? How similar/different are those cysts compared to these of sheep ?

How harmful can be these cysts ? For sheep health? For human health?

What would happen if dogs eat these cysts? What would happen if a person eats these cysts?

**Perception of dogs**

*Positive and negative aspects of dogs*

According to you what are the positive aspects of dogs ? For which reasons dogs are useful or necessary? What are the negative aspects of dogs? In which case they represent a danger or a nuisance ?

*Feeding of dogs*

What dogs are fed with ? Do you feed them yourself ? Where are they looking for their food?

*Stray dogs*

What do you think of stray dogs ? For what are they useful ? For what are they harmful ? How do they impact your village (beside rabies) ?

**Hygiene**

What is the origin of the drinking water for your household?

What is the source of water you use to wash vegetables?

What do you think about the quality of the water you are using to wash vegetables ?

Does it happen sometimes to eat crude vegetables without having washed them before ?

How available is the water to wash your hands before eating? For you ? For your children ?

**Perception of control options**

Imagine that we propose several control measures against echinococcosis (or use local name). What would be the principals obstacles to implement them:

Method 1 : Stop feeding dogs with sheep cysts ?

Method 2 : Feeding of dogs by owners? And their puppies ?

Method 3 : Burying or burning sheep carcass/offal to prevent dogs from eating them ?

Method 4 : Discourage dog ownership ?

Method 5 : Kill stray dogs ?

Method 6 : Replace sheep with goats ?

1. Butchers

**Slaughtering practices and cysts in sheep**

What does your work consist of, and particularly the slaughtering activities ?

What do you keep/throw away ? What do you do with the waste ?

What do you think about the way slaughtered animal waste is currently managed ? How could it be more optimal for you ? What methods would be more appropriate according to you ?

What is missing for improving your work conditions? What is missing for improving slaughter conditions?

(How often do you get veterinary inspections in the slaughterhouses ?

Which organs are controlled?

What does happen with the condemned offal ?

What kind of sanctions are there in case of violations ?)

Do dogs have access to the slaughterhouses ? How do you perceive their presence there ?

How could access of dogs in the slaughterhouses be restricted ? What are the barriers to implement your suggestions ?

*Cysts (Echinoccocus)*

Have you ever found white large cysts on a carcass of a sheep ? On which parts?

How often ?

What did you do when you found these cysts ?

What is the origin of these cysts?

Do you find them also in other animals? How similar/different are those cysts compared to these of sheep ?

How harmful can be these cysts ? For sheep health? For human health?

**Knowledge and perceptions of echinococcosis**

Have you ever heard about cysts in people (men, women, or children) ? What is the name given? Where in the body are these cysts located? Is it frequent in this village ?

How important is this disease in relation to your health? What is the impact on daily life?

What happens when somebody develops such a disease? How do you treat it ? How can you prevent it?

What is the origin of these cysts according to you ?

How can we interrupt the development of these cysts? How can we control the cysts? How do we remove the cysts?

What would happen if dogs eat these cysts? What would happen if a person eats these cysts?

**Economical aspects**

How is the price of a sheep established? Which are the factors that do raise the price or reduce the price ? What is the value of a liver ? How can we ensure that the liver is in good state ?

**Perception of control options**

Imagine that we propose several control measures against echinococcosis (or use local name). What would be the principals obstacles to implement them:

Method 1 : Stop feeding dogs with sheep cysts ?

Method 2 : Feeding of dogs by owners? And their puppies ?

Method 3 : Burying or burning sheep carcass/offal to prevent dogs from eating them ?

Method 4 : Discourage dog ownership ?

Method 5 : Kill stray dogs ?

Method 6 : Replace sheep with goats ?
