# Supplementary material for: Community perception and knowledge of cystic echinococcosis in the High Atlas Mountains, Morocco
Source: BMC Public Health. 2019 Jan 28;19:118. doi: 10.1186/s12889-018-6372-y (PMC6350308; doi:10.1186/s12889-018-6372-y)
Supplement: Supplementary file 2 — Illustrative quotes from the Focus Groups Discussions (a to n). Quotes (a to n) selected from the Focus Group Discussions conducted in the Hight Atlas in Morocco (October - November 2009) to illustrate the results. (DOCX 18 kb) [file 12889_2018_6372_MOESM2_ESM.docx]

**Additional file 2: Illustrative quotes from the Focus Group Discussions (quotes “a” until “n”)**

a : Quote from a focus group of men from the Amizmiz municipality

“*I was saying that the dog did burp in front of its owner who was eating and who had fallen ill just after. Probably that badness is transmitted by the saliva of the dog or just by the smell or the breath coming out of his mouth and which goes through the breath of its owner*”

b : Quote from a focus group of men from the Ourika municipality

*“Only with the smell you can get sick if these things are thrown in the open air.*”

c: Quote from a focus group of butchers from the Aghmat municipality

“*Because this cyst found in sheep (it) is called the dog sickness, and this cyst, this ball of water, if it breaks out, it can be a danger even to the butcher.*”

d: Quote from a focus group with men from the Aït Ourir municipality

“*The animal infected by the cyst will not live, dear Mister (appearing very self-confident). You know, sheep during a heat wave, under the sun until their fat gets hot, and then they go to drink very cold water, here and there in various streams. And well, I tell you, it is what causes cysts.*”

e: Quote from a focus group with men from the Amizmiz municipality

“*Dogs can eat human flesh. Someone from the region of Rhamana had told me one day, and I swear to you, that dogs had attacked a schoolteacher who was traveling on her motorbike. They had devoured her and had left only the hair on the spot.*”

f: Quote from a focus group with men from the Aghmat municipality

*“M2: But if you have a dog you have to take care of it before God*

*M9: Of course*

*M5: These people don't understand, but you know that if you don't take care of him you will be cursed, and you'll be in sin...*

*M2: You will have it on your conscience.*

*M5: You have to give him to eat and take care of him.*

*M2: If you are able to do it, it is good. If not, let him go, free him.*

*M4: And if he doesn't want to leave, you must kill him, right”*

g: Quote from a focus group with women from the Oukaimeden municipality

“*Watching TV, only at night after having finished what we have to do: do the housekeeping, meal preparation, laundry and of course take care of animals by feeding them and get them out to be able to clean the fold.*”

h: Quote from a focus group with butchers from the Aghmat municipality

“*M7: Among us, there are some who dismember, some who slaughter and some who do not dismember...*

*M6: There are some maalems with their apprentices...*

*M7: Bosses with their apprentices...*

*M3: The maalems of the balance, "experts of the meat weighing".*

*M7: And there are wholesalers.*

*M8: There are the skaytiya who deal with offal and resell them.*”

i: Quote from a focus group with men from the Aït Ourir municipality

“*Because finally we (speaking as a butcher) just look at the liver and lungs. If there is a sleazy thing we inform the owner and we remove it, but we are never sure.*”

j: Quote from a focus group with men from the Aghouatim municipality

“*If there are many (cysts), we throw the liver away, if there are few, we remove the infected part, and we keep the rest.*”

k: Quote from a focus group with men from the Oukaimeden municipality

*“(...) because I do not think that dogs eating these cysts will have the illness.*”

l: Quote from a focus group with butchers from the Aït Ourir municipality

“*Bah Yes, we do not remain silent in front of veterinarians, we ask them to explain us the origin of everything. For example for cysts, they tell us it is because the animals live with dogs, and these dogs defecate near animals that can eat it, and animals will also be sick and reject these cysts for dogs.*”

m: Quote from a focus group with students from the Tahannaout municipality

“*Excuse me, a teacher taught us in Islamic education. The sheep slaughtered the day of Eïd must not be eaten meat before 24 hours because the cells are still alive, blood circulation is still functional.*”

n: Quote from a focus group with men from the Amizmiz municipality

“*There are doctors who say that it (cyst) probably comes from food contaminated by the dogs or cats hair...*”
